# Silenced departures: psychometric evaluation of the quiet firing scale among nurses in general hospitals

**DOI:** 10.3389/fpsyg.2026.1762350

**Published:** 2026-05-12

**Authors:** Rui Tang, Shanxia Luo, Sixia Chen, Jie Li

**Affiliations:** 1 Mental Health Center, West China Hospital / West China School of Nursing, Sichuan University, Chengdu, China; 2 Department of Emergency Medicine, Luzhou People's Hospital, Luzhou, China

**Keywords:** nurses, psychometrics, quiet firing, reliability, translation, validation

## Abstract

**Backgrounds:**

Intensifying geopolitical and organizational pressures have amplified psychosocial risks in healthcare, particularly in the context of persistent nursing shortages. These conditions can enable covert managerial behaviors such as quiet firing, which undermine nurse wellbeing and threaten workforce stability. Given the limited empirical evidence, a validated instrument is essential to detect such early risks and inform organizational prevention strategies.

**Method:**

A Chinese version of the Quiet Firing Scale was developed through translation, back-translation, and cross-cultural adaptation. Using purposive sampling, nurses from five general hospitals were surveyed to assess the scale's reliability and validity.

**Results:**

Of 782 returned questionnaires, 720 were deemed valid and included in the psychometric validation. The Chinese version comprised 7 items and demonstrated a unidimensional structure, explaining 67.36% of the total variance in exploratory factor analysis. Item-level content validity indices (I-CVI) ranged from 0.820 to 1.000, with a scale-level CVI of 0.905. Confirmatory factor analysis supported a well-fitting model (CFI = 0.991, GFI = 0.980, NFI = 0.984, IFI = 0.991, TLI = 0.983, RMSEA = 0.060). Convergent validity was confirmed by an Average Variance Extracted of 0.591 and Composite Reliability of 0.894. The scale exhibited strong internal consistency (Cronbach's α = 0.908), split-half reliability (0.939), and test-retest reliability (0.899).

**Conclusion:**

The Chinese version of the Quiet Firing Scale is a psychometrically robust tool for assessing nurses' experiences in general hospitals. It establishes a foundation for examining its prevalence and potential effects on nurse wellbeing and workforce stability.

## 1 Introduction

Heightened geopolitical tensions, economic tightening, and prolonged crises often converge to create complex challenges for organizations (World Health Organization, 2025). During periods of economic uncertainty, budget constraints, and organizational restructuring, many workplaces experience intensified managerial and operational pressures. Although such pressures do not inevitably result in harmful practices, they may increase the likelihood of subtle forms of employee marginalization emerging within organizational systems (Gutiérrez Banegas et al., 2022; Potocan and Nedelko, 2021). Global nursing shortages constitute a long-standing structural problem, exacerbated by an aging workforce and rising turnover rates, and cannot be resolved by individual hospitals alone (Marć et al., 2019; Montayre et al., 2023). Even when patient care demand remains high, hospitals may still reduce nurse staffing due to budgetary and financial pressures (Dierkes et al., 2022). Nursing constitutes the largest single component of hospital labor costs, and hospitals often view nursing departments as cost centers, making them the first to face workforce reductions (Aiken et al., 2014). Budgetary pressures may prompt managers to adopt more stringent or even employee-adverse management practices (Bedford et al., 2022). Among these practices is quiet firing, a subtle yet harmful approach used by some organizations to encourage resignations without implementing formal termination processes.

Quiet firing refers to an employer-driven practice in which organizations, rather than explicitly terminating employment, systematically create hostile or untenable working conditions—through bullying, unfair treatment, unreasonable workload changes, denial of support or opportunities, and other coercive managerial behaviors—that effectively pressure employees, including whistle-blowers, to resign, thereby circumventing legal protections against improper dismissal. While categorized as a form of covert workplace mistreatment, quiet firing is conceptually distinct from other workplace stressors in terms of its functional impact and strategic intentionality. Specifically, quiet firing extends beyond workplace ostracism, which is defined by an individual's perception of being ignored or socially excluded (Howard et al., 2020). Unlike ostracism, which primarily entails a lack of social interaction, quiet firing involves a deliberate and systematic withdrawal of the functional resources, feedback, and professional opportunities essential for a nurse's career survival.

Furthermore, quiet firing is fundamentally strategic and goal-oriented, distinguishing it from workplace bullying and abusive supervision. Workplace bullying involves repeated negative acts aimed at disempowering an individual, and abusive supervision is characterized by a subordinate's perception of sustained hostile verbal and non-verbal behaviors (Mackey et al., 2017; Cowie et al., 2002). Rather than being driven solely by interpersonal hostility or individual personality traits, its primary objective is to induce resignation to mitigate the legal and financial liabilities of formal termination—a phenomenon often legally recognized as constructive dismissal or constructive discharge. Quiet firing erodes healthcare workers' engagement, job satisfaction, and affective organizational commitment (Çevik, 2025). In nursing, it may appear as restricted opportunities for professional development, exclusion from decision-making, or reassignment away from preferred shifts or units. These practices can heighten nurses' intentions to leave their organization and, in some cases, the profession entirely (Othman et al., 2025). Beyond these emerging findings, evidence regarding the impact of quiet firing on nurses and the wider healthcare workforce remains limited.

Measuring quiet firing is crucial because the behaviors involved are typically subtle, undocumented, and difficult to detect through administrative mechanisms. A validated self-report scale captures nurses' subjective experiences, which often represent the earliest warning signs of harmful managerial practices. Reliable measurement also allows examination of the impact of quiet firing on key outcomes such as nurse turnover and patient safety. Moreover, standardized assessment enables healthcare organizations to monitor workplace climate and design timely interventions to prevent escalation and workforce loss.

To address this measurement need, Anand et al. (2024) developed the Quiet Firing Scale in 2023 to assess employees' perceptions of the extent to which managers devalue them and the degree to which organizations intentionally create conditions that encourage resignation. Compared to existing tools measuring workplace mistreatment or turnover intention (Dhanani and Bogart, 2026), the Quiet Firing Scale uniquely focuses on “managerial omission” and “systemic devaluation.” It captures the subtle withdrawal of organizational support—such as strategic exclusion from training or deliberate career stagnation—that remains invisible to traditional administrative audits. Following its initial development in India, the scale's cross-cultural applicability has been further supported by validation studies in Türkiye and Egypt (Çevik, 2025; Othman et al., 2025). These studies reported consistent internal reliability among healthcare personnel and clinical nurses, confirming the instrument's stability and practical utility across diverse clinical settings.

Currently, no validated tool exists to assess quiet firing among Chinese nurses. While high nurse turnover is extensively documented in China, the specific role of managerial agency in driving these separations remains under-explored (Ren et al., 2024). In China's collectivist culture, the emphasis on group harmony often discourages open dismissal. Instead, managers may adopt quiet firing by using indirect behaviors to prompt resignation while maintaining a facade of organizational unity. This phenomenon is further shaped by the coexistence of diverse employment modalities, including permanent, contract-based, and labor dispatch staff. This institutional hierarchy grants managers significant discretionary power to marginalize non-permanent nurses through inequitable resource allocation or career stagnation. To address this gap, this study aims to culturally adapt the Quiet Firing Scale and evaluate its psychometric properties within the Chinese clinical context.

## 2 Methods

### 2.1 Design

This study involved the cross-cultural adaptation and validation of the Quiet Firing Scale, following established guidelines for the translation, adaptation, and validation of instruments in cross-cultural healthcare research (Sousa and Rojjanasrirat, 2011). The study was conducted in two phases: (1) cross-cultural adaptation, and (2) validation of the scale through a cross-sectional descriptive study.

### 2.2 Participants

This study was conducted between March and June 2025 and included nurses currently employed at five general hospitals in western China. Participants were recruited using a convenience sampling method. The inclusion criteria were as follows: (a) nurses engaged in clinical practice at general hospitals, (b) ability to communicate in Chinese, and (c) comprehension of and willingness to participate in the study. Nurses who were on leave, attending external training, or had less than 3 year of work experience (Anand et al., 2024) were excluded. The sample size was determined based on the recommended subject-to-item ratio of 10:1 for factor analysis (MacCallum et al., 1999). Given that the Quiet Firing Scale consists of 7 items, a minimum of 70 participants was required.

### 2.3 Instruments

The first part of the questionnaire collected demographic information, including gender, age, educational background, years of work experience, professional title, and employment type (permanent, contract, or dispatched).

The second part consisted of the Quiet Firing Scale, developed by Amitabh Anand and colleagues, which is designed to assess employees' perceptions of the extent to which their managers devalue them and the degree to which organizations intentionally create situations that encourage them to resign (Anand et al., 2024). The scale is unidimensional and consists of seven items. Each item is rated on a five-point Likert scale ranging from 1 (strongly disagree) to 5 (strongly agree). Total scores range from 7 to 35, with higher scores reflecting a greater perceived level of quiet firing. In the original study conducted among employees in India, the scale demonstrated good internal consistency, with a Cronbach's α of 0.876.

### 2.4 Procedure

Before commencing the study, the researchers obtained permission from the original authors of the Quiet Firing Scale (Anand et al., 2024) to utilize the scale.

#### 2.4.1 Phase 1: cross-cultural adaptation of the quiet firing scale

In the initial stage, a forward–backward translation method was employed. Two independent translations from the original English version to Chinese were conducted: one by a nursing psychology expert and the other by a professional English translator without a medical background. These translations produced two separate Chinese versions, which were subsequently merged to form a consensus version. Following this, the consensus version was back-translated into English by two researchers: a clinical nursing expert and a professional English translator. During the translation and cultural adaptation process, particular attention was paid to items describing subtle managerial disengagement or indirect organizational pressure, as these concepts required careful wording to ensure conceptual equivalence and clarity in the Chinese cultural context. In addition, because the original scale was developed in a corporate context whereas the present study targeted nurses working in hospital settings, minor wording adjustments were made to ensure that the items were appropriate for healthcare organizational environments while preserving the original meaning and structure of the scale.

To ensure the content validity of the adapted Chinese version of the Quiet Firing Scale, a panel of eight experts with experience in psychological research was convened, including two psychology instructors, two nursing education instructors, two nursing managers, and two clinical nursing mentors. A single round of expert consultation was conducted to evaluate the content equivalence and cultural appropriateness of the translated scale. Lawshe's Content Validity Index (CVI), including the Item-level (I-CVI) and Scale-level (S-CVI) indices, was calculated to assess content validity (Lawshe, 1975).

#### 2.4.2 Phase 2: validation of the scale via the cross-sectional descriptive study

A pilot study was conducted among employed nurses using a convenience sampling method. The pilot study aimed to evaluate the cultural comprehensibility and preliminary reliability of the Chinese version. Participants independently completed a preliminary questionnaire administered electronically via the Questionnaire Star (e-questionnaire) platform. Following the survey, individual cognitive interviews were conducted with all participants using “probing” and “paraphrasing” techniques to ensure the items were clear and reflected their clinical experiences. The interviews confirmed that the scale was easy to understand and culturally appropriate. Prior to completing the questionnaire, participants were provided with a detailed explanation of the study objectives and gave informed consent. It was emphasized that participation was entirely voluntary, and that all data would be collected anonymously and kept confidential.

Following the pilot study, the final version of the Quiet Firing Scale was established. A formal descriptive study was then carried out to evaluate the reliability and validity of the scale among nurses in general hospitals in China. Nursing department managers at the participating hospitals were contacted to facilitate recruitment, and participants were provided with the electronic questionnaire along with appropriate incentives. The introduction of the questionnaire clearly outlined the study objectives, significance, participants' rights, and privacy protection measures. After providing informed consent, participants independently completed the questionnaire. Core items of the electronic questionnaire were set as mandatory to ensure completion before submission. To maintain data integrity and prevent duplicate or missing responses, each IP address was allowed to submit only one questionnaire. An exception was made for nurses from a designated tertiary hospital participated in a follow-up assessment 2 weeks after the initial survey to evaluate the consistency of participants' responses.

### 2.5 Ethical considerations

This study was approved by the Ethics Committee of the study hospital (Approval No. 2024, 1907). Written informed consent was obtained from all participants, and the study complied with the principles of the Declaration of Helsinki (World Medical Association, 2001). The research objectives were clearly communicated to all participants. Informed consent forms were duly signed by all participants, who were also informed about the confidential and anonymous handling of their data.

### 2.6 Data analysis

Data analysis was conducted using Excel 2021, IBM SPSS 29.0, and R 4.2.3. Preliminary data screening was performed to ensure data quality. Invalid responses were excluded based on a minimum completion time of 3 min and the presence of identical answers across all items (long-string patterns). Due to the “mandatory response” setting in the electronic questionnaire platform, which prevented submission if any item was left blank, there were no missing data in the collected dataset. The valid sample was randomly split into two independent subsamples for structural validation using the “select cases” function in SPSS. The first subsample was used for exploratory factor analysis (EFA), and the second was utilized for confirmatory factor analysis (CFA). CFA was performed using the lavaan package version 0.6.17 (Rosseel, 2012). Measurement data were presented as means and standard deviations (SDs), while categorical data were reported as frequencies and percentages. Item selection for the scale involved reliability testing, dispersion trend analysis, and critical ratio decision value analysis. Validity analysis encompassed three dimensions: content validity, structural validity, and convergent validity. Content validity was assessed through the CVI (Lawshe, 1975), including Item-level (I-CVI) and Scale-level (S-CVI) indices. Structural validity was evaluated via exploratory and confirmatory factor analyses (Bandalos and Finney, 2018), adjusting the confirmatory model with Modification Indices (MI) as needed. Convergent validity was measured by calculating Average Variance Extracted (AVE) and Composite Reliability (CR) from path coefficients in the confirmatory factor analysis (Cheung et al., 2023). Reliability analysis employed Cronbach's α, split-half reliability coefficient, and Intraclass Correlation Coefficient (ICC). A significance level of *P* < 0.05 denoted statistical significance.

## 3 Results

### 3.1 Cross-cultural adaptation of the quiet firing scale

A single round of Delphi consultation was conducted. The eight experts involved in the study represented multiple fields of expertise (Table 1). Given that several experts possessed dual or multiple specialties, the distribution of their professional backgrounds was as follows: clinical nursing (62.5%), nursing management (62.5%), nursing research (50.0%), and Psychometrics (37.5%). All experts agreed that the items were essential and no items were recommended for deletion. Minor wording adjustments were made based on expert feedback to improve clarity and cultural appropriateness.

**Table 1:** Demographic characteristics of the experts (*N* = 8).

Characteristics	Categories	*n* (%)	Mean ±SD
Gender	Female	7 (87.5)	
	Male	1 (12.5)	
Age (years)			38.63 ± 5.63 (33.0–48.0)
Education level	Master	4 (50.0)	
	Doctor	4 (50.0)	
Professional title	Intermediate	3 (37.5)	
	senior	5 (62.5)	
Years of field experience			14.00 ± 5.77 (7.0–22.0)

### 3.2 Pilot survey

A total of 38 nurses participated in the pilot survey. The preliminary version of the scale demonstrated excellent internal consistency, with a Cronbach's α of 0.848 and a split-half reliability coefficient of 0.845. On average, participants required 3 to 5 min to complete the preliminary questionnaire. Additionally, interviews with the 38 nurses provided valuable insights into their understanding and interpretation of the questionnaire items, confirming that all items were clear, culturally appropriate, and required no further linguistic structural adjustments.

### 3.3 Validation of the quiet firing scale

#### 3.3.1 Participant characteristics

A total of 782 questionnaires were initially collected. After excluding invalid responses, 720 valid questionnaires remained, resulting in an effective response rate of 92.3%. Of these, 622 participants were included in the main psychometric analysis, aged 20.0 to 54.0 years (mean ± SD: 32.16 ± 7.37), 98 participants were invited to complete the questionnaire again after 2 weeks to evaluate test–retest reliability. Among the participants, 93.2% were female (Table 2). All participants reported a mean score of 2.76 ± 0.58 on the Quiet Firing Scale (Table 3).

**Table 2:** Characteristics of the participants (*N* = 622).

Characteristics	Categories	Count	Percentage (%)
Age (Mean ± SD)		32.16 ± 7.37	
Gender	Female	580	93.2
	Male	42	6.8
Education level	Junior college or below	253	40.7
	Bachelor's degree	367	59.0
	Postgraduate degree	2	0.3
Years of work experience	3–10 years	357	57.4
	11–20 years	210	33.7
	≥20 years	55	8.9
Professional title	Junior	380	61.0
	Intermediate	229	36.8
	Senior	13	2.2
Employment type	Labor dispatch	27	4.4
	Contract	480	77.1
	Permanent staff	115	18.5

**Table 3:** Factor loadings and descriptive statistics for items of the Chinese version of the quiet firing scale (*N* = 622).

Items	Factor 1	Mean ±SD
1. My manager/supervisor gives me limited time off from work	0.809	3.05 ± 0.90
2. My manager/supervisor has increased my workload, but no raise or increase in pay	0.844	3.35 ± 0.73
3. My manager/supervisor has demanded to work after hours	0.872	2.63 ± 0.90
4. My manager/supervisor has excluded me or kept me out of the loop in work/social events	0.866	2.33 ± 0.86
5. My manager/supervisor has a lack of respect for my contributions	0.909	2.51 ± 0.90
6. My manager/supervisor fails to give recognition for my performance	0.812	2.73 ± 0.89
7. My manager/supervisor has shown less interest in my career trajectory/development	0.930	2.69 ± 0.80

#### 3.3.2 Content validity

The I-CVI for the Chinese version of the Quiet Firing scale ranged from 0.820 to 1.000, and the S-CVI was 0.905.

#### 3.3.3 Structural validity

**Figure 1:**
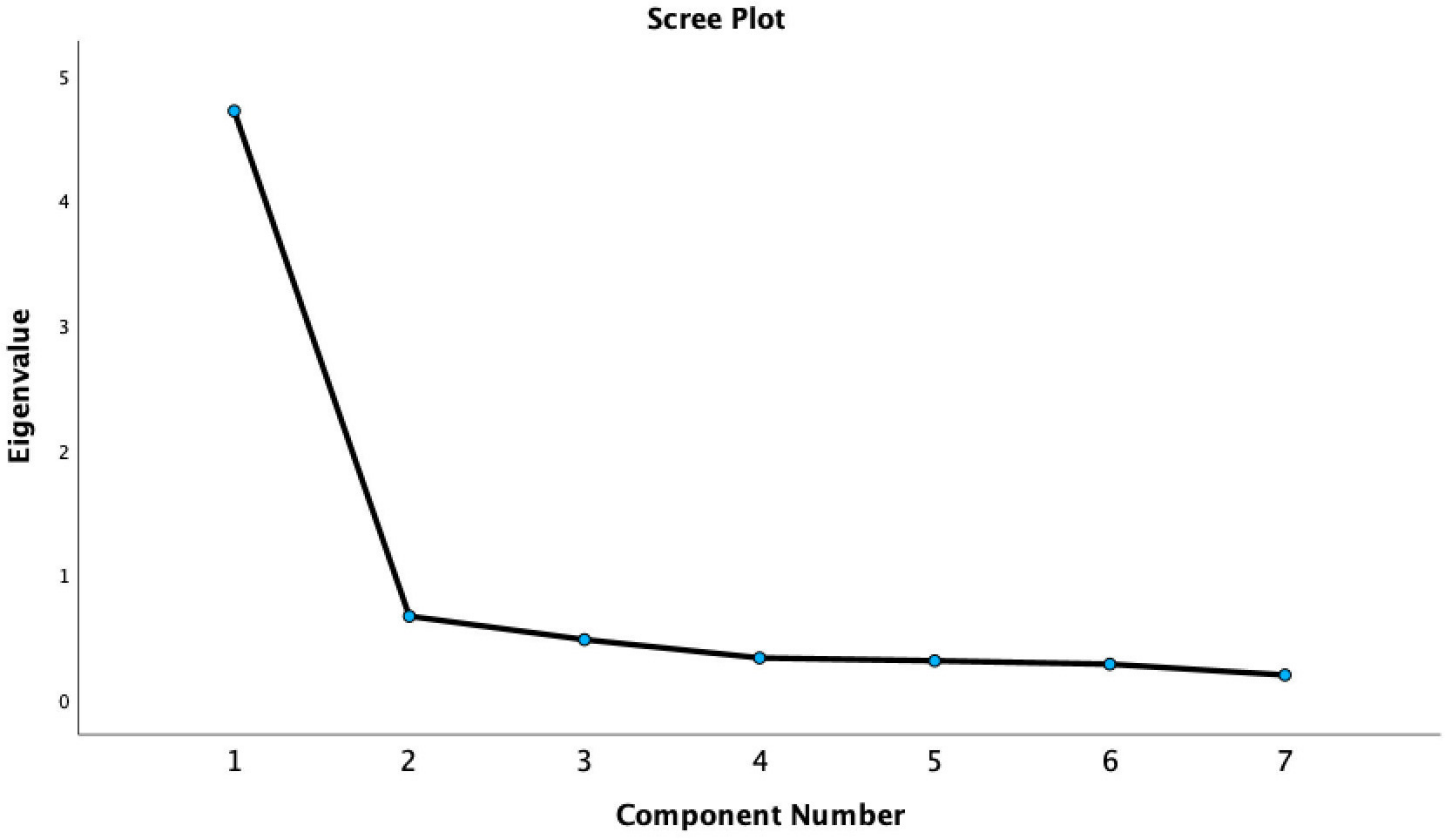
Scree plot of the exploratory factor analysis (*N* = 310).

Six hundred twenty-two nurses were randomly split into two independent subsamples: Subsample 1 (*n* = 310) for EFA and Subsample 2 (*n* = 312) for CFA. EFA was conducted on data from 310 nurses. Bartlett's test of sphericity was significant (χ^2^ = 1437.740, *P* < 0.001), and the Kaiser–Meyer–Olkin (KMO) measure of sampling adequacy was 0.911. Using principal component analysis with Varimax rotation, one single factor with an eigenvalue exceeding 1 was extracted. The cumulative variance contribution rate was 67.36% (Figure 1).

CFA was subsequently performed on data from 312 nurses. The CFA revealed significant correlations among the residuals of Q1 and Q2, Q1 and Q5, and Q5 and Q7. Since these item pairs measure conceptually similar aspects of the latent construct, it is plausible that their measurement errors are associated. Based on MI and theoretical considerations, three correlated error terms were introduced to improve model fit. Specifically, the residuals of Q1 and Q2 were allowed to correlate, as both items reflect the impact of leadership on workload and work conditions, potentially eliciting similar responses. The residuals of Q1 and Q5 were correlated because Q1 (restricted leave) and Q5 (lack of respect) both capture facets of perceived disregard or insufficient support from leadership. Finally, the residuals of Q5 and Q7 were allowed to correlate, as both items pertain to interpersonal neglect and a lack of attention to employees' professional development. These modifications are theoretically justified and preserve the substantive meaning of the latent construct. Following these adjustments, the model demonstrated acceptable fit, with a chi-square to degrees of freedom ratio (χ^2^/*df* ) of 2.135 (*P* < 0.001) and the following fit indices: Comparative Fit Index (CFI) = 0.991, Goodness-of-Fit Index (GFI) = 0.980, Normed Fit Index (NFI) = 0.984, Incremental Fit Index (IFI) = 0.991, Tucker-Lewis Index (TLI) = 0.983, and Root Mean Square Error of Approximation (RMSEA) = 0.060. Overall, these results indicate that the model fits the data well.

#### 3.3.4 Convergent validity

Through confirmatory factor analysis, the path coefficients between each item and the scale ranged from 0.662 to 0.876, indicating strong relationships between individual items and the overall scale. The AVE values for the scale were 0.591, and the CR value was 0.894, demonstrating good convergent validity and scale aggregation.

#### 3.3.5 Reliability analysis

The test–retest reliability of the scale was evaluated with a 2-week interval using a matched sample of 98 participants, yielding a high correlation coefficient of 0.908 (*P* < 0.001). The overall Cronbach's α for the scale was 0.939, and the split-half reliability coefficient was 0.899.

## 4 Discussion

This study adapted and validated the Quiet Firing Scale for use among registered nurses in Chinese general hospitals, aiming to assess their experiences of quiet firing within the nursing workplace. Our findings demonstrate that the Chinese version of the Quiet Firing Scale exhibits strong content, structural, and convergent validity, alongside excellent internal consistency and reliability. The Cronbach's α coefficient was 0.939, exceeding that of both the original scale (0.876) and the Turkish version (0.890). The mean quiet firing score among Chinese nurses (2.76 ± 0.58) was also slightly higher than that previously documented among Egyptian nurses (2.36 ± 0.66) (Othman et al., 2025). The successful cross-cultural adaptation and validation of the Chinese version represent a significant step toward understanding and addressing quiet firing among nurses in Chinese general hospitals, with important implications for nursing management and healthcare practice.

To our knowledge, this is the first study to adapt and validate the Quiet Firing Scale in the Chinese context. The instrument initially developed by Amitabh Anand and colleagues has been widely used to assess quiet firing across multiple occupational groups, including corporate employees, service-sector workers, and early-career academicians, with a Turkish version applied among healthcare staff (Anand et al., 2024; Atiku et al., 2025; Bhawna et al., 2025). Quiet firing has been shown to diminish productivity, generate substantial opportunity costs for both employers and employees, and undermine organizational trust (Oquendo et al., 2026). Given these consequences, and in order to more accurately capture Chinese nurses' perceptions of the extent to which managers devalue them and the degree to which organizations intentionally create conditions that encourage voluntary resignation, a validated measure such as the Quiet Firing Scale is essential for assessing both the nature and severity of quiet firing in this workforce. Our findings are broadly consistent with those reported among Egyptian nurses, although the mean quiet firing score in our sample (2.76 ± 0.58) was slightly higher than the level previously observed in Egypt (2.36 ± 0.66), where a significant positive correlation between quiet firing and turnover intention has already been established (Othman et al., 2025). The elevated scores observed among Chinese nurses may further reflect the insidious impact of this phenomenon on the stability of the healthcare workforce. Although the present study did not examine turnover intention directly, the relatively higher scores in our sample underscore the critical relevance of quiet firing to nursing workforce sustainability and highlight the urgent need for further investigation into its broader organizational consequences.

This study demonstrates that the Chinese version of the Quiet Firing Scale possesses robust psychometric properties. Content validity assessed using the CVI (Minlong, 2010) showed I-CVI values ranging from 0.820 to 1.000, all exceeding the accepted threshold of 0.780, while the S-CVI reached 0.905, surpassing the recommended benchmark of 0.900 (Polit et al., 2007). Regarding structural validity, the exploratory factor analysis identified a single dominant factor accounting for 67.36% of the total variance. The confirmatory factor analysis further supported this unidimensional structure, yielding excellent model-fit indices that met or exceeded recommended criteria (Perry et al., 2015). Convergent validity was confirmed by strong item loadings (0.662–0.876) and satisfactory AVE and CR values, all of which were above standard thresholds (Minlong, 2009). The reliability analysis demonstrated strong internal consistency, with coefficients exceeding those reported for both the original English version and the Turkish adaptation. These findings indicate that the scale shows excellent stability when applied among nurses in Chinese general hospitals. Our findings are broadly consistent with those reported among Egyptian nurses, although the mean quiet firing score in our sample (2.76 ± 0.58) was slightly higher than the level previously observed in Egypt (2.36 ± 0.66) (Othman et al., 2025). In particular, Chinese nurses reported elevated scores on aspects related to restricted access to leave (3.05 ± 0.90) and increased workload without corresponding compensation (3.35 ± 0.73), both of which exceeded the overall mean score. These patterns may reflect the persistent staffing shortages, high patient loads, and limited autonomy in work scheduling that characterize many Chinese hospital settings, all of which have been shown to contribute to increased work pressure and reduced perceived organizational support among nurses (Al-Hakim et al., 2022; Aubé et al., 2007; Pursio et al., 2021).

The present study provides an important foundation for understanding quiet firing in healthcare settings, a phenomenon that remains relatively underexplored despite its potential organizational and workforce implications. The development of a validated scale offers nursing managers and healthcare organizations a systematic instrument for identifying subtle forms of managerial disengagement that may otherwise remain difficult to detect. Establishing a reliable measurement tool represents an essential first step, enabling researchers to assess the prevalence of quiet firing, examine its potential antecedents, and inform the development of targeted interventions aimed at safeguarding both care quality and workforce sustainability. Routine assessment using this scale may enable hospital administrators to identify departments in which nurses feel undervalued or perceive organizational conditions that implicitly encourage voluntary resignation. Such insights may facilitate more targeted managerial responses, including leadership development initiatives, improved workload allocation strategies, and efforts to strengthen communication between frontline staff and supervisors. At present, the scale is primarily intended to assess the relative level of quiet firing experiences, with higher scores indicating greater perceived exposure to such practices, as no established cut-off point is currently available in either the original or adapted versions. Future research may therefore seek to determine clinically or organizationally meaningful threshold values to support interpretation and practical application. Although quiet firing may not always lead to negative outcomes, future research may use this instrument to examine the associations between quiet firing and key workforce indicators, such as patient safety outcomes, nurse psychological wellbeing, burnout, and job satisfaction. Longitudinal studies will be particularly valuable in clarifying whether quiet firing functions as an early organizational signal preceding voluntary turnover or declining work engagement among nursing staff.

This study has several limitations. First, participants were recruited through purposive sampling from comprehensive hospitals in Western China, which may restrict the generalizability of the findings to other regions or different levels of healthcare institutions. Future research should employ multi-center, random sampling to enhance external validity. Second, as the survey was distributed online and participation was voluntary and anonymous, information on individuals who declined participation was not available; therefore, the characteristics of non-responders could not be analyzed. Third, while the structural validity and reliability were robust, this study did not assess criterion-related validity by correlating the scale with objective outcomes such as turnover intention or job satisfaction. Establishing these associations remains a priority for future longitudinal studies. Fourth, the reliance on self-report questionnaires may introduce social desirability bias, although the anonymity of the survey was emphasized. Finally, although the psychometric analyses provide initial evidence of reliability and validity, further evaluation in broader clinical contexts and larger, more heterogeneous samples is needed to strengthen the robustness of the scale.

## 5 Conclusions

This study provides a rigorously cross-culturally adapted and psychometrically sound Chinese version of the Quiet Firing Scale for use among nurses in general hospitals. The findings demonstrate that the instrument exhibits strong content validity, structural validity, convergent validity, and excellent reliability, supporting its suitability for assessing nurses' experiences of quiet firing in the Chinese healthcare context. As the first validated tool to quantify quiet firing in Chinese nursing settings, the scale offers a valuable instrument for identifying subtle forms of managerial disengagement that may influence nurses' perceptions of organizational support and professional value. The availability of this measurement tool establishes an important foundation for future research to investigate the prevalence, antecedents, and potential organizational consequences of quiet firing. Further studies are warranted to explore its associations with key workforce outcomes, including patient safety outcomes, nurse wellbeing, burnout, job satisfaction, and turnover intention, and to inform evidence-based management strategies aimed at promoting workforce sustainability and improving healthcare organizational environments.

## Data Availability

The raw data supporting the conclusions of this article are available from the corresponding author upon reasonable request. Requests to access the data should be directed to the corresponding author.
